# Set up and assessment of progression criteria for internal pilots: the Brushing RemInder 4 Good oral HealTh (BRIGHT) trial example

**DOI:** 10.1186/s40814-023-01243-z

**Published:** 2023-01-27

**Authors:** Hannah Ainsworth, Zoe Marshman, Katie Whiteside, Debbie Sykes, Caroline Fairhurst, Emma Turner, Ivor Chestnutt, Peter Day, Donna Dey, Louise Elliott, Sarab El-Yousfi, Catherine Hewitt, Claire Jones, Sue Pavitt, Mark Robertson, David Torgerson, Nicola Innes

**Affiliations:** 1 York Trials Unit, Department of Health Sciences, University of York, York, UK; 2 School of Clinical Dentistry, University of Sheffield, Claremont Crescent, Sheffield, UK; 3 School of Dentistry, Cardiff University, Heath Park, Cardiff, UK; 4 Cardiff and Vale University Health Board, Cardiff, UK; 5 School of Dentistry, University of Leeds, Leeds, UK; 6 Bradford Community Dental Service, Bradford District Care NHS Foundation Trust, Bradford, UK; 7 School of Education and Social Work, University of Dundee, Nethergate, Dundee, UK; 8 Health Informatics Centre, University of Dundee, Dundee, UK; 9 School of Dentistry, University of Dundee, Park Place, Dundee, UK

**Keywords:** Dental caries, Caries prevention, Behaviour change, Internal pilot, Progression criteria, Randomised controlled trial, Child dental health, mHealth, Short messaging service

## Abstract

**Background:**

Dental caries is common in young people and has wide-ranging ramifications for health and quality of life. Text messaging interventions show promise as a means to promote oral health behaviour change among young people. This paper reports the internal pilot of the Brushing RemInder 4 Good oral HealTh (BRIGHT) trial, which is evaluating an intervention comprising an oral health classroom lesson and text messages about toothbrushing, on caries in young people. Pilot trial objectives were to evaluate the feasibility and appropriateness of recruitment and data collection methods, the randomisation strategy, and intervention delivery against progression criteria for the main trial.

**Methods:**

This is an internal pilot trial embedded within an assessor-blinded, two-arm, cluster randomised controlled trial. Participants were pupils aged 11–13 years (in year 7/S1 or year 8/S2) in secondary schools in England, Scotland, and Wales with above average pupil eligibility for free school meals. Following completion of pupil baseline questionnaires and dental assessments, year groups within schools were randomised to the intervention or control arm. Approximately 12 weeks later, participants completed a follow-up questionnaire, which included questions about sources of oral health advice to assess intervention contamination between year groups. At the end of the pilot phase, trial conduct was reviewed against pre-specified progression criteria.

**Results:**

Ten schools were recruited for the pilot, with 20 year groups and 1073 pupils randomised (average of 54 pupils per year group). Data collection methods and intervention delivery were considered feasible, the response rate to the follow-up questionnaire was over 80%, there was an indication of a positive effect on self-reported toothbrushing, and interest was obtained from 80% of the schools required for the main trial. Despite partial intervention contamination between year groups, within-school randomisation at the level of the year-group was considered appropriate for the main trial, and the sample size was revised to account for partial contamination. Facilitators and barriers to recruitment and data collection were identified and strategies refined for the main trial.

**Conclusions:**

Progression to the main trial of BRIGHT, with some design refinements, was concluded. The internal pilot was an efficient way to determine trial feasibility and optimise trial processes.

**Trial registration:**

ISRCTN registry, ISRCTN12139369, registered 10/05/2017

**Supplementary Information:**

The online version contains supplementary material available at 10.1186/s40814-023-01243-z.

## Key messages regarding feasibility

- What uncertainties existed regarding the feasibility? The following uncertainties regarding feasibility existed: feasibility of school and participant recruitment, data collection methods, within-school randomisation (level of contamination), and embedding the education component of the trial intervention (classroom lesson) within the curriculum.
- What are the key feasibility findings? All the above-mentioned uncertainties were found to be feasible, with some study modifications.
- What are the implications of the feasibility findings for the design of the main study? Continuation to the main trial was confirmed with the study design based on within-school randomisation. The sample size (originally based on between-school randomisation) was revised, and some study modifications were made to optimise recruitment and data collection strategies.

## Background

Dental caries in young people is common, affecting an estimated one in three 12 years old in the UK and rising to almost one in two among 12–15 years old living in deprived areas [1]. Observational studies have shown that the frequency, duration, and efficacy of toothbrushing are inadequate in young people [2, 3]. Poor oral health can have significant and wide-ranging consequences for the health and quality of life of children, including impaired cognitive development, poor school attendance, and difficulty with schoolwork [2, 4]. Treating dental disease costs NHS England £3.4 billion per year, with children’s tooth extractions accounting for £50.5 million a year [5]. There is a clear need for effective behavioural interventions that encourage adherence to caries prevention recommendations among children and adolescents [6].

The use of mobile phones, in particular Short Message Service (SMS) or text messaging, has been studied as a means to promote positive health behaviour change among young people, though more rigorous research on this is required [7]. A non-randomised, longitudinal study of unemployed 18–24 years old in New Zealand investigated the use of the Keep on Brushing programme (involving regular motivational SMS messaging and free toothbrushes and toothpaste) on toothbrushing [8]. The study found that self-reported twice (or more)-daily toothbrushing increased from 51% at baseline to 73% 9 weeks later, indicating that such an intervention shows promise and warrants investigation in a larger-scale randomised controlled trial (RCT).

In the UK, it is estimated that 93% of 12–15 years old own their own smartphone [9], and research suggests that smartphone ownership among children does not vary by socio-economic status [10]. Therefore, interventions using such technology may have potential for achieving behaviour change in this population. To date, there is a paucity of research looking at the use of mobile phones as a means to deliver behaviour change interventions in teenagers. Due to this, we developed an intervention adapted from the one used in the Keep on Brushing study and aimed to evaluate the clinical and cost-effectiveness of this intervention within the Brushing RemInder 4 Good oral HealTh (BRIGHT) trial. Full details can be found in the trial protocol [11] and a paper discussing development of the intervention [12]. In brief, the objectives of the BRIGHT trial were as follows:

1. Conduct an internal pilot trial.
2. Investigate the effect of the intervention on caries prevalence.
3. Investigate the effect of the intervention on twice-daily tooth brushing, oral health-related quality of life, and oral health behaviours.
4. Investigate the cost-effectiveness of the intervention.
5. Explore implementation, mechanisms of impact, and context through a process evaluation.

The focus of this paper is the internal pilot within the BRIGHT trial. There are two types of pilot studies: external and internal [13]. External pilot, or feasibility, studies are generally recommended when there is greater uncertainty over the likely success of a full-scale efficacy or effectiveness (main) trial. They are completed and analysed externally to a main trial, and the participants and associated data do not contribute to the main trial. Amongst others, reasons to conduct an external pilot trial are to determine intervention and study feasibility, to inform sample size calculations, and test outcome measures before further costly evaluation. Successful external pilots may then lead to larger trials, but there is a gap between the two as this often requires a new funding application. Internal pilots, however, are more suited to situations where there is less uncertainly about the success of a main trial and where key components of the intervention and outcome measures are unlikely to change, since participants and the associated data collected within an internal pilot are included with data collected as part of the main phase of the trial in order to achieve the required sample size [13–15]. Researchers often specify a particular time frame (e.g. first 6 months of recruitment), or percentage of the recruitment window, or the point at which a certain number of participants have been randomised, to constitute the internal pilot phase of the trial, at the end of which progress is often judged against pre-specified criteria, often relating to recruitment (site and participant), retention, and intervention delivery rates [13]. If these are met, the trial continues to completion, occasionally with small adjustments to procedures to improve trial conduct.

In a recent review of National Institute for Health Research (NIHR) health technology assessment (HTA)-funded trials with an internal pilot phase, 63% of trials were found to have specified progression criteria in the latest available version of their protocol [14]. More frequent and transparent reporting of internal pilot trials as standard has been called for in order to increase understanding amongst researchers and inform future trials, as well as to clearly detail decision-making around the progression criteria [13, 14]. This paper therefore aims to report the BRIGHT internal pilot, clearly presenting the findings associated with each progression criteria, detailing decision-making, and providing justification and study modifications for the main trial.

The objectives of the BRIGHT internal pilot, described in this paper, were to use pre-defined criteria to determine progression to the main trial phase, including an exploration of potential contamination and therefore the appropriateness of the trial design, assessment of the required sample size, potential for positive effect and feasibility of delivery of the intervention, recruitment of schools and participants, and data collection methods. As far as possible, we report in line with the CONSORT 2010 extension for randomised pilot and feasibility trials, and the checklist is provided (Additional file 1), though it should be noted that the extension does not directly apply to internal pilot studies [15].

## Methods

### The BRIGHT trial

The BRIGHT trial is a school-based, assessor-blinded, two-arm, cluster RCT [11]. The East of Scotland Research Ethics Committee provided ethical approval for the trial (REC reference: 17/ES/0096). In brief, the BRIGHT intervention involves a short (approximately 50 min), teacher-delivered, classroom-based lesson on dental health, which can be embedded into UK secondary schools’ personal, social, health and economic (PSHE) education programmes. This is followed by a series of twice-daily, personalised SMS messages to encourage toothbrushing. Pupils in the control arm receive neither the lesson nor the text messages. The primary outcome for the overall trial is caries prevalence for obvious decay experience at approximately 2.5 years, defined as the presence of at least one treated or untreated carious lesion in any permanent tooth, measured at the pupil level using the DMFT (decayed, missing, and filled teeth) index, where decay is measured as carious lesions extending into dentine (International Caries Detection and Assessment System [ICDAS] levels 4–6 [16]). The presence of caries is measured during clinical assessments conducted in participating schools at baseline and at either 2 or approximately 2.5-year follow-up. It should be noted that since the publication of the protocol paper [11], amendments have been approved to alter the outcome follow-up time points for logistical reasons. For example, the published protocol states that the final follow-up will be at 3 years; however, this was amended to 2.5 years to avoid clashing with GCSE examination periods in schools. The most recent protocol is available on the website of the funder — the NIHR HTA [17].

At the outset, the BRIGHT trial planned to recruit 48 secondary schools in England, Scotland, and Wales, of which 10 were to be recruited during the internal pilot phase. This sample size was based on between-school randomisation. At least four clusters per arm are recommended for cluster pilot RCTs [18]; therefore, 10 schools were planned to be included in the internal pilot, to meet this recommendation and to accommodate for potential withdrawal of schools.

Eligible schools were identified based on data from the Department for Education’s register of educational establishments in England [19] and Welsh and Scottish government websites [20, 21]. For the pilot phase, purposive sampling from eligible school lists for South Yorkshire, West Yorkshire, and South Wales was conducted, and schools were approached through email, letter, or phone call. The research teams in these regions also made use of existing contacts with schools and engaged with local educational organisations to recruit schools. In Scotland, schools were introduced to the trial and invited to take part in the pilot phase at a meeting for Scottish head teachers. These strategies were used to mitigate for the short time frame for recruitment and maximise engagement and feedback on how to improve processes for recruitment of schools to the main trial phase.

To be eligible to take part, schools had the following:

- Be located in Scotland, South Wales, or England (South Yorkshire and West Yorkshire)
- Be state funded
- Have pupils aged 11–16 years old
- Have at least 60 pupils per year group
- Have an above national average percentage (in 2016, for each devolved nation) of pupils who were eligible for free school meals (FSM)

Schools were ineligible if they were as follows:

- Judged, at the time of recruitment, by the Office for Standards in Education, Children’s Services and Skills (Ofsted) as ‘requires special measures’ (i.e. were judged to be failing to provide pupils with an acceptable standard of education) [22]
- Due to close

Within-school randomisation (which would require a smaller sample size) was used in the internal pilot phase in order to explore the feasibility of this approach for the main trial phase. For the pilot, year groups within schools (n=20) acted as the ‘clusters’ (i.e. the unit of randomisation). Allocation took place within schools by randomising schools 1:1 to one of two regimes: (1) pupils of 11–12 years (year 7 in England and Wales/S1 in Scotland) to receive the intervention and pupils of 12–13 years (year 8 in England and Wales/S2 in Scotland) to act as the control group or (2) pupils of 12–13 years (year 8 in England and Wales/S2 in Scotland) to receive the intervention and pupils of 11–12 years (year 7 in England and Wales/S2 in Scotland) to act as the control group. An allocation sequence, stratified by school using blocks of size two, was generated by an independent York Trials Unit statistician. Once baseline assessments were complete for a school, the year groups in that school were randomised by allocating them to the next available block in the sequence in the order year 7/S1 and then year 8/S2. The year groups allocated to the intervention were then asked to deliver the lesson on oral health, after which the twice-daily SMSs to pupils were commenced.

The internal pilot trial began recruitment of pupils at the start of the 2017/2018 academic year and was designed to inform the main trial which was to begin recruitment the following academic year.

Seven progression criteria were developed by the trial team and agreed (based on the commissioning brief and guidance from the funder NIHR HTA) (Table 1). At the pre-specified time point in the study, which was set at June 2018, they were to be considered by the Trial Management Group, the independent Trial Steering and Data Monitoring and Ethics Committees, and the funder before continuation to the main phase.

**Table 1:** Progression criteria

Progression criteria		Outcome
1	Recruitment of an average of 48 pupils per year group from the schools included in the pilot phase (48 being 80% of the target average recruitment of 60 pupils per year group)	Achieved, some study modifications planned
2	Confirmation of the feasibility of the outcome data collection methods and time points within the school year	Achieved, some study modifications planned
3	Minimum of 80% response rate to questionnaires at follow-up	Partially achieved, some study modifications planned
4	Assessment of contamination in the control group and whether feasible and more efficient to continue randomisation within schools (by year group) or switch to randomisation at the school level in the main phase of the trial and calculation therefore of the required number of schools	Within-school randomisation was feasible. Sample size recalculated
5	Agreement to participate in principle obtained from 80% of the number of schools required for the main trial (based on 4)	Achieved
6	Confirmation of feasibility of embedding the education component within the curriculum	Achieved
7	An indication of a positive effect of the intervention on self-reported frequency of tooth brushing, at approximately 12-week follow-up, using a one-sided 80% confidence interval approach	Achieved

### Progression criterion 1 — recruitment of participants

Pupils aged 11–13 years old attending a participating secondary school in years 7 and 8 (England and Wales), and years S1 and S2 (Scotland), with their own mobile telephone, were eligible to take part in the BRIGHT trial. The aim was to recruit approximately 60 pupils in years 7/S1 and 60 pupils in years 8/S2, giving a total of 120 pupils per school.

Members of the research team visited each school and delivered information sessions to explain the BRIGHT trial to pupils (typically within assemblies). Information packs for parent/carers and pupils were developed with input from patient and public involvement (PPI) work supported by the charity ‘Children and Young People’s Empowerment Project’ (Chilypep) and a panel of parents. The parent/carer packs were sent via the participating school to the parent/carers of all pupils in these years. The packs included a parent/carer information sheet, a copy of the pupil information sheet and consent form, and a parent/carer opt out form. The parents/carers were given 2 weeks to consider their child’s participation in the BRIGHT trial and could decline by completing and returning the opt-out form to the school. If no opt-out form was received, it was assumed they were happy for their child to make their own decision to participate.

All eligible pupils whose parents/carers had not opted them out of the BRIGHT trial were invited to take part and were provided with the pupil information pack. They were given 2 weeks to consider whether they wanted to participate and, if so, were asked to sign a consent form and complete a contact form to provide their mobile telephone number and preferred timings for the text messages. If they could not provide a valid mobile phone number, they became ineligible.

Data on participant recruitment rates were collected, alongside feedback from local teams as to the barriers and facilitators to recruitment of participants. Progression criterion 1 would be achieved if an average, across all 10 of the schools, of 48 pupils per year group were recruited. This would equate to 80% of our target of 60 pupils per year group.

Following an information sessions to pupils, BRIGHT trial information packs (cover letter signed by the school head teacher, a parent/carer information sheet, parent/carer opt-out form, and a copy of the pupil information sheet and consent form) were distributed to the parents/carers of all pupils in participating classes via post or by sending them home with pupils. Parents/carers could decline their child’s participation by completing and returning the opt-out form to their child’s school within a 2-week opt-out window. Schools were requested to record which pupils had been opted out on a spreadsheet. If parents/carers did not return an opt-out form within the 2-week window, it was assumed they were happy for their child to decide themselves if they would like to participate. Parents/carers could withdraw their child at any point over the trial. Eligible pupils were provided with a pupil information sheet and asked to complete a consent form if they agreed to take part. Schools were requested to do this within class or form time in a dedicated consent session and LRTs offered to deliver or facilitate these sessions. As the information session would have taken place at least 2 weeks prior to the consent session (i.e. before the parent/carer opt-out window), pupils were able to consent to take part within the consent session. Schools were requested to make additional pupil information sheets and consent forms available for any pupils who were absent on the day of the consent session or who wanted more time to consider participation. Completed consent forms were checked to ensure parents/carers had not opted them out. Pupils completed a contact form with their mobile telephone number and to indicate their text message preference times and preferred name to be used in the text messages should they be in the intervention group. If they did not own their own mobile telephone or could not provide their own mobile telephone number, they were considered ineligible for participation.

### Progression criteria 2 and 3 — feasibility of outcome data collection methods and response rates

#### Baseline data collection

Data on pupils’ levels of dental caries were collected at baseline by trained and calibrated dental teams via assessments carried out in schools using standard dental epidemiology protocols based on the National Dental Epidemiology Programme in England [23] and the National Dental Inspection Programme (NDIP) Scotland [24]. Pupils were also asked to complete a two-part questionnaire investigating frequency of toothbrushing and oral health behaviours (using validated questions from the national Children’s Dental Health Survey) [1, 25], toothbrush and toothpaste availability, child oral health-related quality of life (CARIES-QC) [26], and general health-related quality of life (CHU-9D) [27]. PPI work by Chilypep facilitated the refinement of the questionnaires for pupils.

#### Follow-up data

**Fig. 1:**
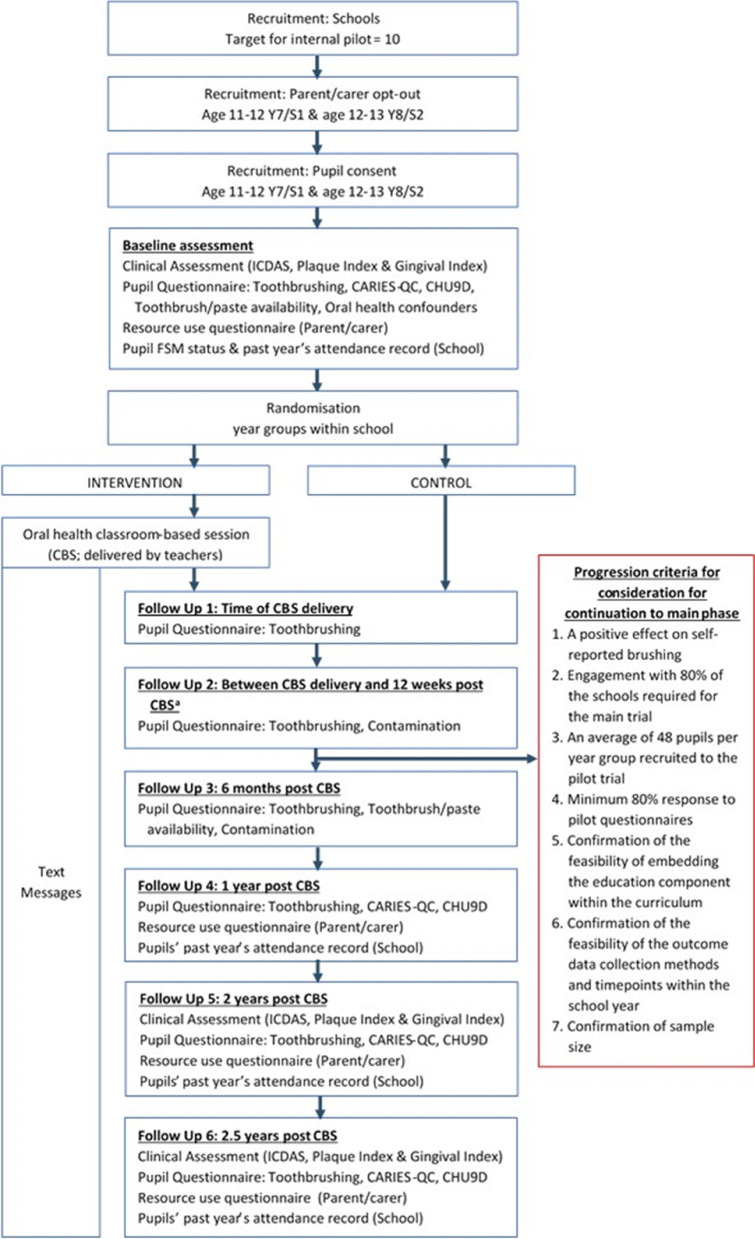
Internal pilot trial design diagram. ^a^Where it was not possible to conduct the first two follow-ups for the internal pilot before progression criteria review due to time constraints, pupils were asked to complete just one follow-up only to reduce burden on schools and pupils. This was completed between the time of the CBS and 12 weeks and included the question on contamination, which was required for the progression review. The exact time point for each school depended on the time available before the progression criteria review. *ICDAS*, International Caries Detection and Assessment System. *CARIES-QC*, Caries Impacts and Experiences Questionnaire for Children (child oral health-related quality of life). *CHU9D*, Child Health Utility-9D (child health-related quality of life)

The internal pilot originally included two follow-up time points; planned to be reached before a decision to progress to the main trial would need to be made, in June 2018, before recruitment to the main trial began in August 2018. Follow-up time point 1 was immediately after the lesson on oral health and involved a questionnaire collecting data on frequency of toothbrushing and oral health behaviours. Follow-up 2 was planned to take place 12 weeks after the lesson to collect data from pupils via a questionnaire on frequency of toothbrushing and oral health behaviours, plus whether, and from where, they had received helpful information about how to keep their teeth and mouth healthy (to assess for potential contamination in the control group, discussed further in ‘Progression criterion 4— randomisation and between-arm contamination’). However, due to delays in recruitment and baseline data collection (discussed further below), and in order to collect as much useful data as possible before consideration of the progression criteria, a decision was taken to stop collecting the follow-up 1 questionnaire from some schools and only collect follow-up 2 data (to ensure that pupils were asked the question related to contamination, which was only included in the follow-up 2 questionnaire). The timing of follow-up 2 was altered so that it could be collected immediately after the lesson had been delivered or anytime up to 12 weeks later. Figure 1 lays out the trial timeline.

The completion rate for the pupil dental assessment at baseline and the return rate for the pupil questionnaires at baseline, follow-up 1, and follow-up 2 was calculated.

### Progression criterion 4 — randomisation and between-arm contamination

At the outset of this trial, we considered two options for the unit of randomisation: (i) school and (ii) year group. It was more efficient to randomise at the level of the year group, meaning a smaller number of schools is required to participate, than if we randomised at the level of the school (both year groups within the school would be allocated to the same trial arm), to detect the same intervention effect. However, a disadvantage of this approach is the increased risk of contamination. Contamination was considered to be participants in the year group allocated to the control arm of the trial receiving some of the intervention; this was possible as there were pupils within the same school as them receiving it. For example, the school may accidently deliver the lesson to the wrong year group, or control pupils may hear intervention participants discussing the text messages and decide to look into receiving something similar themselves through other means. When this happens, the control arm becomes more similar to the intervention arm, and there is a risk that the observed intervention effect will be diluted. A high level of contamination could actually have rendered within-school randomisation the less efficient option.

In the pilot trial, we randomised at the year-group level with the option of moving to randomisation at the level of the school for the main trial phase if this proved infeasible, unacceptable, or there was a high level of contamination. The feasibility and acceptability of randomising by year group within schools were considered by the trial management group, local research teams (LRTs), and through communication with participating schools.

To assess contamination, the follow-up 2 questionnaire for pupils asked the following:

‘Have you received helpful information about how to keep your teeth and mouth healthy from any of these places?’

A variety of potential sources were then listed, of which the ones relevant to the BRIGHT intervention were a lesson in school, friends in another year group, and text messages. Pupils in the control group selecting these responses might indicate some contamination. We recalculated the sample size after the pilot trial assuming the same level of contamination found in the pilot trial was likely to continue in the main trial. If this sample size, in terms of number of schools, was still lower than it would be based on school-level randomisation, then we would retain randomisation at the year group level; otherwise, we would switch to school level.

### Progression criterion 5 — engagement and recruitment of schools

Progression criterion 5 stated that we should have engagement (obtain agreement to participate, at least in principle) with 80% of the number of schools required for the main trial by the end of the pilot, based on the revised sample size. In addition to the school recruitment strategies employed for the pilot trial, others used for the main trial included using contacts made during pilot recruitment, use of personal contacts and contacts held by the recruiting universities and local councils, advertising through local authority networks, contacting Academy Trust chief executives, involving local school nursing teams, and through head teachers recommending the trial to other head teachers. Feedback from local teams was captured on the barriers and facilitators to recruitment.

### Progression criterion 6 — feasibility of embedding the education component within the curriculum

This was explored through discussion with teachers involved in the internal pilot trial and consideration of the current curriculum requirements in each of the devolved nations.

### Progression criterion 7 — effect of the intervention on self-reported toothbrushing

The issue of estimating treatment effects in pilot or feasibility studies is controversial, since they are not usually designed or powered to formally assess evidence of effectiveness. However, it may be of interest to consider if an intervention shows preliminary evidence of benefit in a pilot trial to inform the decision, amongst other factors, to conduct, or continue to, a confirmatory trial [28]. This estimation could be based on the primary clinical endpoint, or instead, it may be based on a surrogate outcome, perhaps one measured at an earlier time point, which reliably predicts the clinical outcome [29].

In this pilot trial, pupils were asked at follow-up point 2 how often they brushed their teeth. At least twice-daily tooth brushing is a key secondary outcome in the BRIGHT trial, while the primary outcome is dental caries. We hypothesise that success on the primary outcome will be mediated through an increase in adequate tooth brushing as this will provide remineralising fluoride through application of toothpaste and also remove potentially cariogenic dental plaque biofilm. Therefore, given this was a large and costly trial, we wanted some assurance that the intervention had promise in this population, at least early on in the follow-up when the oral-health messages from the lesson were still fresh in the minds of intervention participants and they were likely to still be receiving the text messages. Therefore, progression criterion 7 was judged as met if there was an indication of a positive effect of the intervention on self-reported frequency of toothbrushing at follow-up 2 using a one-sided 80% confidence interval (CI) approach, as recommended by Cocks and Torgeson [30]. Their approach recommends planning the sample size for a pilot trial such that it is large enough that if there is no difference between the intervention and control group, then the one-sided 80% CI for the treatment effect would be narrow enough to exclude (i.e. the upper limit would be lower than) a chosen minimum clinically important difference. This way, if the upper one-sided 80% confidence limit was under the pre-specified minimum difference, you could be reasonably confident that the intervention does not show sufficient potential benefit.

We aimed to recruit 1200 pupils from 10 schools within the pilot phase. Assuming 60 pupils per year group, 20% attrition, and an intra-cluster correlation coefficient (ICC) of 0.02, this sample size would be sufficient to produce a one-sided 80% CI for the difference in proportion of pupils who report twice-daily toothbrushing that excludes 5% in the event of a zero (or negative) effect of the BRIGHT intervention, assuming 66% reported brushing twice-daily in each of the two groups [30, 31].

To determine whether the intervention increased the likelihood of pupils brushing twice-daily, the proportion of pupils who reported at least ‘twice-daily’ brushing, as opposed to ‘never’ or ‘once a day’, was compared between the two groups using a binary logistic multilevel model, adjusting for school year (year 7/S1 or year 8/S2) as a fixed effect covariate and school as a random effect. The treatment effect in the form of an odds ratio, and adjusted risk difference, and associated one-sided 80% CI would be presented.

## Results

A summary of the progression criteria outcomes is given in Table 1.

### Progression criterion 1 — recruitment of participants (an average of 48 pupils per year group required for main trial progression)

**Fig. 2:**
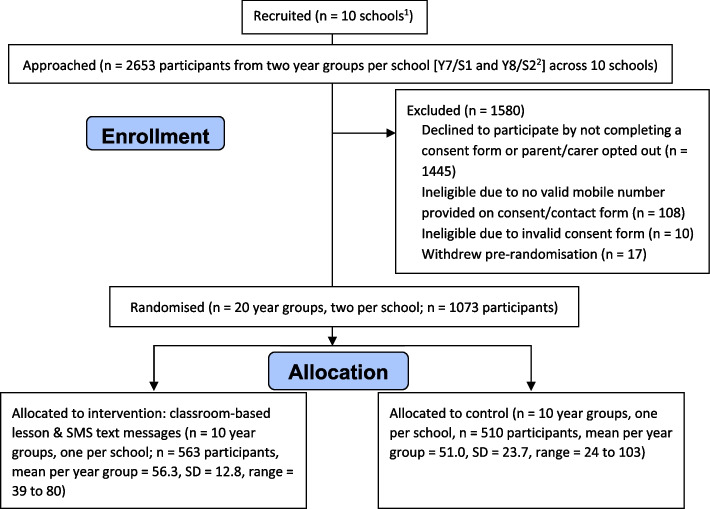
CONSORT diagram for the internal pilot phase. ^1^Eleven schools were actually recruited in the pilot, but two of the recruited schools were due to merge in the 2018/2019 academic year (single-sex schools merged into two mixed sex schools) and therefore are considered as one school for the purposes of the trial (i.e. they have been randomised as one school). ^2^Year 7 (age 11–12) and year 8 (age 12–13) were approached in schools in England and Wales, and S1 (age 11–12) and S2 (age 12–13) were approached in schools in Scotland

In total, 1090 pupils consented to the trial; 17 withdrew before randomisation, resulting in 1073 pupils randomised into the trial from 10 schools (Table 2, Fig. 2). Progression criterion 1 was therefore met, as we recruited an average of 54 pupils per year group.

**Table 2:** Pupil recruitment in the pilot phase

	Target to randomise	Invited	Consented (% of invited)	Randomised (% of target)
**Average per year group**	60	133	55 (41%)	54 (90%)^a^
**Total across all schools**	1200	2653	1090 (41%)	1073 (89%)

^a^The target average recruitment was 60 pupils per year group, and the progression criteria required reaching 80% of this, so an average of 48 pupils per year group across all schools

This was, however, still lower than the 60 we hoped to achieve. We were able to calculate an estimate of the participation rate for the number of pupils approached: an average of 133 pupils per year group was invited to take part in the trial, and 54 (41%) were randomised (see Table 2). We were therefore satisfied that we could achieve an average of 60 recruited pupils per year group in the main phase by approaching a larger pool of pupils in each year group (i.e. by inviting, on average, at least 150 pupils per year group) and adopting other changes to recruitment strategies and solutions to potential barriers (see Tables 3 and 4).

**Table 3:** Pupil recruitment strategies

Recruitment strategy	Successful or unsuccessful	Decision regarding implementation in the main trial phase
Raising the profile of the BRIGHT trial within the school environment through local research teams presenting at assemblies	Successful	Continue in all schools
Maximising researchers’ presence in schools and being available to answer questions from pupils directly	Successful	Continue, with a commitment to researcher flexibility and multiple visits to schools if required
Continuing to recruit pupils during baseline data collection at the school, when pupils could hear feedback from participating peers	Successful	Continue
Working with school staff to smooth pathways for recruitment, e.g. school staff allowing time during class to complete consent forms	Successful	Continue
6.2.1. Provision of appropriate information to parents/carers where English was an additional language for them, where possible	Not required for schools in pilot	Provide if requested by schools in main trial
Informing pupils that they would receive a ‘Thank you’ voucher on completion of baseline data	Successful	Continue
Inviting a random selection of classes within large year groups to avoid over recruitment	Unsuccessful: Typically, the recruitment target was not met with this approach	Invite whole year groups at outset to make best use of resources and to decrease time taken to reach sample size. Random sampling of recruited pupils could be used if there was over recruitment
Informing pupils that they would be entered into a ‘Thank you’ prize draw (where they could win an additional £100 in vouchers) on completion of baseline data	Unsuccessful: LRTs fed back that this appeared not to be appreciated by potential participants	Not to be used

**Table 4:** Barriers to recruitment

Recruitment barriers	Solution for the main trial phase
In some schools, higher than expected proportions of pupils did not have a mobile telephone (meaning they did not meet eligibility criteria for participation)	No solution possible
Pupils sometimes struggled to accurately complete the combined consent and contact form, leading to a high number of queries, which took time to resolve	Consent form and contact form separated for the main trial to streamline the consent process. Contact form completed at the time of baseline data collection, so a researcher can give assistance to pupils and support completion and reduce errors
Pupils were sometimes unable to remember their mobile telephone number and were not permitted to access their mobile telephones on school premises due to school rules	Researcher available on school site to support pupil completion. Consent form and contact form separated. Contact form completed outside of usual classroom situation, with permission from school senior leadership for pupils to access mobile phone to check number
Pupils sometimes struggled to understand the language used in the consent forms, despite best efforts to make the consent form appropriate for pupils (e.g. some pupils did not understand the word ‘signature’)	Further simplification of the forms was undertaken. Further PPI review by Chilypep. Researcher available on school site to support pupil completion
The two optional consent statements on the paper consent form appeared to make completing the consent form more complicated or confusing for pupils	The optional consent statement giving permission to be contacted for future projects was removed. The optional consent statement relating to permission for future data linkage with routine data sources was made non-optional, to be consistent with all other consent statements. All consent statements on the consent form had to be initialled or marked to indicate agreement in order for the consent form to be valid
Delays caused by other competing demands in school, such as Ofsted inspections, which lead to researchers’ planned days to be onsite to support recruitment being postponed or schools planned sessions for pupils to complete consent forms being postponed	No solution possible
Changes to the leadership or organisation of schools lead to barriers in organising planned session for recruitment	LRTs to endeavour to keep in close communication with identified key contacts
A 2-week window for pupils to consent, after the parent/carer 2-week opt-out window had passed, delayed the recruitment process	As pupils heard about BRIGHT in an assembly before the opt-out window, and information was sent home, an additional 2-week window was deemed unnecessary. Therefore, the requirement to wait 2 weeks for pupils to consent after the parent/carer opt-out window was dropped for the main trial

Through regular team meetings and discussion with schools, we identified successful pupil recruitment strategies in the pilot to take forward into the main phase of the trial and unsuccessful strategies to drop, thereby making efficient use of resources (Table 3). Barriers to recruitment were also identified, with possible solutions to address them for the main phase (Table 4).

### Progression criteria 2 and 3 — feasibility of data collection methods and response rates (feasibility demonstrated and minimum 80% response rate required for main trial progression)

Following feedback from schools and the LRTs, the data collection methods and follow-up time points within the school year were deemed to be feasible, and progression criterion 2 was judged to have been met. A number of facilitators that maximised data collection during the pilot phase were identified to continue using in the main trial phase (Table 5). Completion of baseline data collection did, however, take longer than anticipated. Barriers for data collection were identified, and solutions were considered (Table 6).

**Table 5:** Data collection facilitators

Facilitator	Decision regarding implementation in the main trial
‘Thank you’ vouchers to pupils following completion of the baseline assessments	Continue
The provision of an appointment schedule for the dental assessments and use of a researcher to collect pupils from class	Continue
Use of two dental teams (when possible) in school, to minimise time in schools and potential disruption for schools	Continue when possible
The presence of researchers in schools during data collection time points to assist with managing trial paperwork, answer queries about the trial, and support completion of the young person questionnaires (e.g. for help with reading or comprehension)	All local research teams to offer to go in to support follow-up data collection in schools, even at time points not involving clinical assessments
Asking pupils to complete their baseline questionnaire while waiting for their dental assessment	Continue, as opposed to holding separate questionnaire completion sessions
Asking pupils to complete the two parts of the baseline questionnaire together to save time. This had initially been divided into two parts to make it less overwhelming for pupils to complete and to allow it to be completed in two sittings if necessary	Combine into one questionnaire as completing both parts of the questionnaire at the same time was acceptable to pupils

**Table 6:** Data collection barriers

Barriers	Solution for the main trial
Some schools allowed pupils to take follow-up questionnaires home, rather than complete during class time, and this strategy was associated with lower response rates	Further encourage schools to ensure follow-up questionnaires are completed during school time, e.g. registration
There was strong feedback from schools to avoid any data collection in the summer term so that clashes with examinations could be avoided	The timelines for each school were bespoke depending on their enrolment date and when the pupils were randomised. The timelines for each school, however, took into account the necessity to avoid times that were considered high stakes by the schools while still fitting in with the timelines required for the trial
Difficulties organising mutually agreeable dates for baseline dental assessments between the schools and LRTs	LRTs to try to complete a timeline of planned trial activities with the school, including planned dates for baseline data collection, at trial entry
Impact of a spell of severe weather conditions, e.g. resulting in school closures	No solution possible
Dental assessments require space and privacy and therefore the need for a room to be available in school for the duration of data collection which was not timetabled for other use	This was achieved by careful discussions with senior management teams — experience from pilot trial informed how we approached schools and what we asked for in main trial

Progression criterion 3 was a minimum of 80% response to questionnaires at follow-up. It was judged to have been partly met; among schools that had available follow-up data at the progression criteria review point, 71% of pupils completed follow-up 1, and 80% completed follow-up 2 (Table 7). The lower response rate at follow-up 1 was driven by two schools that had substantially lower response rates than the other schools that completed this follow-up due to sending the questionnaires home with pupils, rather than asking them to complete them in school time. This was noticed after monitoring follow-up 1 response rates, and schools were subsequently reminded that questionnaires are to be completed in school time.

**Table 7:** Baseline and follow-up pupil questionnaire completion

	N randomised	Baseline dental assessment	Baseline questionnaire part 1	Baseline questionnaire part 2	FU1 questionnaire (time of lesson)^**a**^	FU2 questionnaire (between time of lesson and 12 weeks)^**b**^
***N*** **(% of randomised, % of randomised and asked to complete**^**c**^**)**	6.2.2. 1073	1029 (95.9, N/A)	1030 (96.0, N/A)	1029 (95.9, N/A)	421 (39.2, 71.2)	523 (48.7, 80.1)

*FU* follow-up. ^a^At the time of reviewing the progression criteria, follow-up 1 data was available from 6 pilot schools. ^b^The average time point of follow-up 2 was 9 weeks after the lesson. At the time of reviewing the progression criteria, FU2 data was available from 7 pilot schools. ^c^Some schools were asked not to complete FU1 but to only complete FU2 due to time constraints. Response rate was calculated for 591 pupils for FU1 (i.e. all randomised pupils in the six schools that FU1 data were available for) and 653 pupils for FU2 (i.e. all randomised pupils in the seven schools that FU2 data were available for). These were the only schools in which pupils were asked to complete FU1 and FU2, respectively, by the progression criteria review point

### Progression criterion 4 — randomisation and between-arm contamination (feasible and more efficient to continue randomisation within schools (by year group) despite some contamination, required for main trial progression without design change)

We were conservative in our original sample size calculation, planning, and costing for the worst-case scenario, in case it was necessary to use the less efficient design of randomisation at the school level. The estimated proportion of the UK 12 years old with caries was 32%, with estimates rising to 46% for those eligible for FSM compared to 30% for those not eligible [1, 32]. Based on a systematic review of interventions for caries prevention to increase the frequency of tooth brushing, a reduction of caries prevalence of 8% might be expected [33]. An individually randomised trial powered at 90% (5% two-sided *α*) to detect an 8% absolute reduction, from 32 to 24%, in caries would require 1320 pupils. Few estimates of school-level ICCs are available for dental data. In a previous study evaluating a behaviour change programme for preventing dental caries in primary schools, an ICC of 0.01 was used which was estimated using their own unpublished data (Pine et al., 2016) [34]. Assuming school level randomisation, using a more conservative ICC of 0.02, assuming an average of 60 consented pupils per year group (120 per school), and allowing for 20% attrition, we calculated 48 schools would be required in total (5760 pupils). We noted that should within-school randomisation prove feasible, then assuming within-school randomisation and no contamination, and with all other assumptions as above, we estimated 30 schools (3600 pupils) would be required.

At follow-up 2, we collected information on whether pupils had received helpful information about how to keep their teeth and mouth healthy from the following: a lesson in school, friends in another year group, and text messages. Overall, of the pupils allocated to the control arm who provided a response to the follow-up 2 questionnaire, 63.6% said they had received oral health messages from at least one of these sources (Table 8). This proportion was mainly driven by 58.5% responding that they had received helpful oral health messages from a lesson at school. However, we are aware of only one school that (in error) provided the classroom lesson to the control year group. Given the wording of the question *‘*Have you received helpful information about how to keep your teeth and mouth healthy from any of these places?’, it is possible that pupils responded in relation to any point in their lives rather than just since the beginning of their participation in the trial. They may also have interpreted discussion of the BRIGHT trial in assemblies or form classes as ‘receiving helpful information about how to keep your teeth and mouth healthy’. When we considered only the pupils who said they had received oral health messages from friends in another year group and/or text messages (Table 8), and those in the school where the control year received the classroom lesson, the potential contamination rate in the usual care group was 27%. Even then, we considered it unlikely that all 27% received the full intervention effect as they were unlikely to have received the classroom lesson and be receiving twice-daily SMS tooth brushing reminders.

**Table 8:** Control group relevant responses to the contamination question at follow up 2 (FU2)

FU2 question 15: have you received helpful information about how to keep your teeth and mouth healthy from any of these places?	Control group (***n*** = 272) ***n*** (%)
A lesson in school	159 (58.5)
Friends in another year group	23 (8.5)
Text messages	29 (10.7)
Any of the above	173 (63.6)

We revised the sample size calculation incorporating the contamination effect and also the within-school randomisation. Assuming partial contamination effects (i.e. those contaminated gain half the treatment benefits) for 27% of the control participants, 40 schools would be required in total across the pilot and main trial phases, assuming within-school year group level randomisation, an ICC of 0.02, an average of 60 pupils per year group, and 20% attrition at follow-up. This would give 90% power (5% two-sided *α*) to detect an 8% absolute reduction, from 32 to 24%, in the proportion of pupils with caries. We therefore concluded that within-school randomisation remained the more efficient design choice for the main trial, over switching to school-level randomisation. (Note, we ultimately decided to recruit 42 schools to mitigate against potential whole-school drop-out.)

### Progression criterion 5 — engagement and recruitment of schools (80% of schools recruited, of target for main trial, required for main trial progression)

In addition to the 10 schools recruited during the internal pilot phase, we required at least 30 more schools to be recruited in the main trial phase, based on the revised sample size calculations. We had obtained interest or agreement to take part from 24 (80% of 30) schools at the progression criteria review point; therefore, progression criterion 5 as originally outlined was met.

Through regular team meetings, sharing of information between LRTs and in discussion with schools, a number of school recruitment strategies were found to be successful and were continued while recruiting the remainder of the schools required for the main phase (Table 9). Schools often had competing demands (e.g. high staff turnover, high absentee rates, or competing priorities, such as a focus on improving attainment), which presented a barrier to participation, as taking part in dental research was viewed as a low priority. Also, not all schools had dedicated staff who could approve and sign the data-sharing agreement (DSA), or some schools required DSAs to be signed by local authority colleagues. DSAs are now considered necessary for the nature of data sharing required in a study such as BRIGHT, and as such, this can present an additional barrier to recruitment of schools.

**Table 9:** School recruitment strategies

School recruitment strategy	Successful or unsuccessful
Use of personal contacts based in schools	Successful
Use of school contacts held by recruiting universities	Successful
Asking schools to recommend the study to other schools	Successful
Engaging local or national educational organisations such as local authorities and teacher groups’ educational events	Successful
Making use of local authority education networks for head teachers and senior management teams	Successful
Communicating with academies with several schools in local area	Successful
Face-to-face meetings with school staff	Successful
Approaching schools who had already taken part in similar research studies, such as the children’s dental health survey and a smoking prevention study [35]	Successful
Involving local school nursing teams	Successful
Making contact with schools via letter from the local principal investigator	Successful
Making initial contact with schools via email	Unsuccessful: Schools rarely responded to emails from unknown researchers. Not relied upon for further recruitment

Due to the availability of school-level data and the expanse of the regions eligible to participate, it was not feasible to calculate the number of schools excluded due to not meeting various inclusion criteria (e.g. having at least 60 pupils per year group) or due to meeting the exclusion criteria. Local regional teams checked school eligibility where they could before directly approaching individual schools and checked the eligibility of each school that expressed interest in participating regions against the school inclusion and exclusion criteria. Due to the recruitment methods used in some regions, which reached an unknown number of schools (e.g. advertising through local authority networks and through local or national organisations for schools, such as School Leaders Scotland), it also was not possible to calculate the number of schools approached across all regions.

### Progression criterion 6 — feasibility of embedding the education component within the curriculum (feasibility demonstrated required for main trial progression)

As part of the LRTs contact with the schools in the internal pilot, through discussion and informal feedback, it was found that although schools had different arrangements for the provision of PSHE, it was feasible to embed the lesson into the school’s curricula. The need for flexibility was highlighted, for example lesson delivery over 2 or 3 shorter sessions rather than one 50-min session. Very positive feedback was received on the quality of the lesson plan, including on content, duration, and level of interactivity. Progression criterion 6 was therefore judged to have been met. Further detail on the acceptability of the BRIGHT intervention can be found in ElYousfi et al. (2021) [36]. Recent government guidance now requires oral health to be included as part of the curriculum [37, 38]. However, at the time of the internal pilot, this was not the case, though many school staff were aware of its upcoming inclusion in the curriculum and were therefore grateful for the BRIGHT education component.

### Progression criterion 7 — effect of the intervention on self-reported tooth brushing (indication of positive effect required for main trial progression)

At follow-up 2, 246/296 pupils (83.1%) in the intervention group and 213/272 pupils (78.3%) in the control group reported that they brushed their teeth at least twice a day (difference of 4.8 percentage points in favour of the intervention group; adjusted odds ratio 1.32, upper one-sided 80% confidence limit 1.59). This equates to an adjusted risk difference of 4.2% (upper one-sided 80% confidence limit of 10.7%). The intervention effect is positive, and the upper limit exceeds 5% so we can be reasonably confident that the intervention group was more likely to brush their teeth twice a day than the control group; thus, sufficient preliminary evidence of a treatment difference was observed to support the continuation of the trial.

## Discussion

This paper reports the internal pilot of the BRIGHT trial. The ‘Results’ section above provides a detailed report against each of the pre-defined study progression criteria. Although not all the criteria were entirely met, a decision to progress to main trial phase with some study modifications (also outlined in detail in results section) was taken by the study team, with the support of the independent oversight committees (Trial Steering Committee and Data Monitoring and Ethics Committee) and funder (NIHR HTA). Detailed consideration of each of the progression criteria both alone and in combination allowed for the context and possible explanations for outcomes/findings to be fully considered and study modifications to be proposed.

Indeed, in any study, the decision to progress (or not) to the main trial phase is unlikely to be made on the basis of one particular criterion alone, as others have previously described [13]. At the time of defining the progression criteria, a traffic light approach was not widely recommended and was therefore not adopted; however, it has advantages, which have been discussed more fully recently [39] and in this case may have further aided decision-making at the review point. Under the current NIHR HTA guidelines for progression criteria [40], 100% recruitment of sites (in this case — schools) and participants is required to meet green level; the recruitment levels set (and achieved) as part of the BRIGHT progression criteria are more closely aligned with current amber level examples.

The final design of the main phase of the BRIGHT trial was dependent upon the results of the internal pilot, which found evidence of only minimal between-year group contamination (27%); randomisation at the year group level therefore continued to be implemented, as this had the following advantages:

- It was more efficient in regard to sample size than randomising at the school level (even accounting for contamination), which reduces overall costs of the study, by, for example, reducing the amount of data collection required and the length of time required for recruitment.
- It had greater perceived benefits for schools, in that at least half of their participating pupils receive the intervention. Recruitment and retention of schools in the pilot trial were based on this model; it may have proved more difficult had school level randomisation been adopted.

Given the feasibility of within-school randomisation, but the evidence for some level of contamination, based on data collected in the internal pilot, it was appropriate in this case to revisit the original sample size. It was determined that 40 schools would be required in total for the BRIGHT trial, and the study team took the decision to aim to recruit 42 schools in total to account for any whole school dropout during the main phase of the trial.

The internal pilot also provided valuable information on how the study should be conducted going forward, allowing the study team to make refinements to the school and participant recruitment strategies and providing increased certainly that recruitment targets could be met within revised timeframes. For example, in the main phase of the trial, the default strategy was for whole year groups to be invited to participate from the outset, to ensure the required sample size could be met more quickly. Simplifications to consent and data collection procedures, and revisions to time points, were also made based on learning from the internal pilot. For example, the pupil consent procedure was changed so that pupils’ mobile phone numbers were not collected at the time of consent but at the point of baseline data collection. This allowed the LRTs to better support pupils with providing this important information correctly. The prize draw of a £100 voucher for pupils who agreed to participate was removed, as this did not appear to support recruitment. Changes were also made to the pupil questionnaires for main trial phase, combining the baseline questionnaire into one for simplicity, rather than in two parts.

No major changes to the BRIGHT intervention or proposed outcome measures were highlighted by the internal pilot as being necessary, and therefore, trial data from the internal pilot and main trial participants can be combined at final analysis as anticipated. Overall, the BRIGHT study will therefore benefit from the advantages of conducting an internal pilot, including the cost savings of immediate study continuation.

## Conclusions

Internal pilot trials can provide a time and cost-efficient way to determine intervention acceptability and study feasibility before progression to a main (efficacy or effectiveness) trial phase. However, they are not currently widely reported; indeed, there are currently no CONSORT guidelines for reporting internal pilots. Where they are reported, there is often a focus on the acceptability of the intervention, but issues of trial feasibility, suitability of trial design and decisions in relation to progression criteria are rarely discussed. This paper reports on an example of an internal pilot embedded in a secondary school-based dental trial. It outlines the pre-defined study progression criteria and reports outcomes against these. It describes how the pilot trial phase informed and resulted in optimisation of the recruitment, retention, and data collection strategies to be used during the main phase of the trial, as well as how the feasibility of within-school randomisation was explored and the unit of randomisation confirmed for the main trial phase. It highlights the advantages of conducting internal pilots, as well as lessons learnt to inform future trials.

### Limitations

A purposive approach to recruiting schools to the pilot was adopted, which may have meant participating schools were more enthusiastic about taking part in research than schools who would take part in the main trial.

A traffic light approach to progression criteria may have aided decision-making at the review point. Some allowance for the criteria being partially met, e.g. ‘amber’ in the traffic light system, would have made reporting a little clearer.

### Recommendations

At study design, researchers should consider the appropriateness and potential advantages of integrating an internal pilot into the study design, particularly in situations where there may be uncertainty around intervention and/or study feasibility.

Researchers should prespecify and publish progression criteria as part of routine publication of study protocols and should consider a traffic light approach and/or holistic approach to progression criteria review.

Barriers and facilitators to recruitment and data collection should be carefully recorded and considered to inform meaningful study modifications, giving the main trial the best chance of success.

Researchers should publish data from internal pilots and throw light and transparency on the decision-making process around trial continuation, for the benefit of the wider research community.

Researchers should use learning from pilot trials to improve the efficiency of running the main study. In this study, for example by altering the consent processes and making them more appropriate for the children and schools participating, we reduced errors in data collection, especially for key data such as the pupils phone numbers.

## Supplementary Information

**Additional file 1.**

## Data Availability

The datasets used and/or analysed during the current study are available from the corresponding author on reasonable request.

## Abbreviations

BRIGHT: Brushing RemInder 4 Good oral HealTh trial
Chilypep: Children and young people’s empowerment project
CONSORT: Consolidated Standards of Reporting Trials
DenTCRU: Dental Translational and Clinical Research Unit
DMFT: Decayed, missing, and filled teeth
DSA: Data-sharing agreement
FSM: Free school meals
HTA: Health technology assessment
ICC: Intra-cluster correlation coefficient
LRT/s: Local research team/s
NHS: National Health Service
NIHR: National Institute for Health Research
Ofsted: Office for Standards in Education, Children’s Services and Skills
PHSE: Personal, social, health and economic education
PPI: Patient and public Involvement
RCT: Randomised controlled trial
REC: Research ethics committee
SMS: Short Message Service
UK: United Kingdom
